# Modified Pathway to Survival highlights importance of rapid access to quality institutional delivery care to decrease neonatal mortality in Serang and Jember districts, Java, Indonesia

**DOI:** 10.7189/jogh.13.04020

**Published:** 2023-04-14

**Authors:** Henry D Kalter, Philip W Setel, Poppy E Deviany, Sri A Nugraheni, Sri Sumarmi, Emily H Weaver, Kamaluddin Latief, Tika Rianty, Fitri Nandiaty, Trisari Anggondowati, Endang L Achadi

**Affiliations:** 1 Institute for International Programs, Department of International Health, Johns Hopkins Bloomberg School of Public Health, Baltimore, Maryland, USA; 2 Vital Strategies, New York, New York, USA; 3 Center for Family Welfare, Faculty of Public Health, Universitas Indonesia, Depok, Indonesia; 4 Faculty of Public Health, Diponegoro University, Semarang, Indonesia; 5 Faculty of Public Health, Airlangga University, Surabaya, Indonesia; 6 Carolina Population Center, The University of North Carolina at Chapel Hill, Chapel Hill, North Carolina, USA

## Abstract

**Background:**

Three-quarters of births in Indonesia occur in a health facility, yet the neonatal mortality rate remains high at 15 per 1000 live births. The Pathway to Survival (P-to-S) framework of steps needed to return sick neonates and young children to health focuses on caregiver recognition of and care-seeking for severe illness. In view of increased institutional delivery in Indonesia and other low- and middle-income countries, a modified P-to-S is needed to assess the role of maternal complications in neonatal survival.

**Methods:**

We conducted a retrospective cross-sectional verbal and social autopsy study of all neonatal deaths from June through December 2018, identified by a proven listing method in two districts of Java, Indonesia. We examined care-seeking for maternal complications, delivery place, and place and timing of neonatal illness onset and death.

**Results:**

The fatal illnesses of 189/259 (73%) neonates began in their delivery facility (DF), 114/189 (60%) of whom died before discharge. Mothers whose neonate’s illness started at their delivery hospital and lower-level DF were more than six times (odds ratio (OR) = 6.5; 95% confidence interval (CI) = 3.4-12.5) and twice (OR = 2.0; 95% CI = 1.01-4.02) as likely to experience a maternal complication as those whose neonates fell fatally ill in the community, and illness started earlier (mean = 0.3 vs 3.6 days; *P* < 0.001) and death came sooner (3.5 vs 5.3 days; *P* = 0.06) to neonates whose illness started at any DF. Despite going to the same number of providers/facilities, women with a labour and delivery (L/D) complication who sought care from at least one other provider or facility on route to their DF took longer than those without a complication to reach their DF (median = 3.3 vs 1.3 hours; *P* = 0.01).

**Conclusions:**

Neonates’ fatal illness onset in their DF was strongly associated with maternal complications. Mothers with a L/D complication experienced delays in reaching their DF, and nearly half the neonatal deaths occurred in association with a complication, suggesting that mothers with complications first seeking care at a hospital providing emergency maternal and neonatal care might have prevented some deaths. A modified P-to-S highlights the importance of rapid access to quality institutional delivery care in settings where many births occur in facilities and/or there is good care-seeking for L/D complications.

Neonatal mortality (NM) in 2018 constituted 47% of the global 5.3 million under-5 years (U5) deaths [1], and nearly 80% of neonatal deaths occurred during the early neonatal (EN) period of the first seven days of life [2]. Pregnancy and labour and delivery (L/D) complications are the most important risk factors for perinatal mortality ((PM) = stillbirths plus EN deaths) [3-8], with care provided during L/D affording the greatest reductions in NM [9,10] and prevention of stillbirth [10]. Delivering in a health facility has been shown to decrease the risk of PM due to intrapartum complications by 43% to 58% [8], with higher-level facilities capable of providing basic (BEmONC) and comprehensive (CEmONC) emergency obstetric and newborn care best positioned to deliver the intrapartum care needed to contribute to maternal, perinatal, and neonatal survival [9,10].

Over the past three decades, institutional delivery in Indonesia progressively increased from 18% of all live births in 1989-1994 to 40% in 1998-2002, reaching 75% in 2013-2017 [11-16], but with only 32% of births still taking place in hospitals [16]; now, the government seeks to extend hospital coverage to remote and underserved areas [17]. During the same time period (1990-2017), Indonesia’s neonatal mortality rate decreased by 52%, from 31 deaths per 1000 live births in 1990 [18] to 15 per 1000 in 2013-2017 [16]. By 2017, Indonesia had nearly achieved Sustainable Development Goal 3.2.2, which is to reduce NM in all countries to at least as low as 12 per 1000 by 2030 [19]. Further NM reduction by Indonesia will depend on its success in decreasing early neonatal mortality (ENM), which fell by 44% from 1990-2017, compared to the 52% decrease in NM during the same period [11,16].

These trends were paralleled globally. Over the two decades prior to 2013-2018, the percentage of institutional deliveries increased by 46% globally, with the largest increases in regions with initially low levels such as West and Central Africa (49%) and South Asia (132%). By 2013-2018, 76% of all births worldwide, including 58% in sub-Saharan Africa and 72% in South Asia, were in a health facility [20]. Simultaneously, from 1990-2018, NM decreased globally by 51%, from 37 to 18 deaths per 1000 live births, and in all world regions from 35%, from 48 to 31 per 1000, in West and Central Africa to 71%, from 21 to 6 per 1000 in Eastern Europe and Central Asia [18]. Meanwhile, the share of neonatal deaths due to ENM increased from 71% to 80% during that time [2].

We conducted the Every Mother and Newborn Counts (EMNC) study of maternal and neonatal deaths in Serang and Jember districts, Java, to inform policy, planning, and intervention selection to combat maternal and neonatal mortality in Indonesia. Estimates of the causes of neonatal deaths in Indonesia are based on statistical models [21]; and while pregnancy and delivery complications and delivery without a skilled attendant have been identified as risk factors for NM [22,23], we found just one study from Indonesia on care-seeking for severe neonatal illness in the era of increased institutional delivery [24] and none on care-seeking for maternal complications in the context of neonatal death. To fill these gaps, we aimed to investigate neonatal causes of death and, for the identified deaths, to examine maternal care, care-seeking for maternal complications, and care-seeking for neonatal illness along the Pathway to Survival (P-to-S) framework (Figure S1 in the **Online Supplementary Document**) [25] developed to support the implementation of the WHO/UNICEF Integrated Management of Childhood Illness (IMCI) approach [26].

The Pathway depicts household and community preventive measures meant to maintain health, and care-seeking needed to return sick young children to health [27], and has proven to be a useful model in settings where many or most children die at home [28,29]. However, it was not designed to examine preventive maternal care and care-seeking for sick ENs. Given the high rate of institutional delivery, the strong association of maternal complications with PM, and the fact that most neonatal deaths occur during the EN period, we hypothesized that most neonatal deaths are among children whose mothers had a complication and whose fatal illnesses began in their delivery facility (DF). In support of Indonesia’s and global efforts to identify and monitor the impact of appropriate policies and interventions against NM, we sought to develop an extended P-to-S that highlights maternal care and mothers’ response to complications and to examine mothers’ and their fatally ill neonates’ steps along this Pathway. We previously examined care-seeking for sick neonates and neonatal causes of death [30]. We now focus on maternal complications, mothers’ care-seeking for their complications, delivery place, and association of the complications with newborns’ place of illness onset and severity.

## METHODS

### Study population

We described the study populations and methods extensively elsewhere [30]. Briefly, this was a retrospective cross-sectional study that examined all neonatal deaths from June to November 2018 in Serang District of Banten Province and July to December 2018 in Jember District of East Java Province, both in Java, Indonesia. We selected the six-month recall period based on the need to minimize this potential source of bias in a study that required detailed information, including on the chronology of care-seeking actions taken by the mother for herself and her newborn. We selected the districts based on the representativeness of their provinces in terms of maternal and neonatal mortality levels, quality of health facilities and local government commitment, population size, and mix of urban, rural, and remote communities [31]. We used the Neonatal Deaths from Informant/Neonatal Deaths Follow On Review (NODE-IN/NODE-FOR) double capture method adapted from a similar method for maternal deaths [32] to identify and list possible neonatal deaths. We asked local informants most knowledgeable about vital events in the community to list all known neonatal deaths and stillbirths, together with the parents’ contact information and age at death of neonates. We conducted follow-up interviews with the families to determine which cases met the study eligibility criteria, including that the child was born alive, was of gestational age ≥28 weeks, died on day 0-27 following birth, died during the recall period, and their parents being residents of one of the study districts.

### Interview instrument and definitions

We conducted a verbal and social autopsy (VASA) interview with the main caregiver (usually the mother) of each eligible neonate using a questionnaire adapted from the Johns Hopkins Institute for International Programs VASA instrument [27]. The instrument contains the 2016 World Health Organization verbal autopsy (VA) form [33], which includes questions on maternal complications, neonatal illness signs and symptoms, and the place of birth and death. Social autopsy questions, including those needed to identify maternal antenatal care (ANC) and care-seeking for maternal complications and neonatal illnesses, are interspersed chronologically with the VA questions throughout the questionnaire.

We examined pregnancy (before labour onset) and L/D complications defined by algorithms of illness signs and symptoms (box 1). We defined “Any complication” as having one or more of preeclampsia/eclampsia, prolonged rupture of membranes (PROM), antepartum haemorrhage (APH), fever during labour, prolonged labour, malpresentation or cord complication. We defined care-seeking delays as the time to decide to seek care (“delay 1”) and to reach the DF after deciding to seek care (“delay 2”) for the mother’s L/D symptom(s). We defined delivery place as “delivery hospital” (DH), “lower-level delivery facility” (LLDF) and “community” (home, on route to a health facility, other place). Only hospitals in Indonesia can be accredited as CEmONC facilities, while only the highest level LLDFs (puskesmas: community health centers overseen by the Ministry of Health) can be BEmONC facilities. We were not able to determine which hospitals and LLDFs in our sample were CEmONC- and BEmONC-capable or accredited.

Box 1 **Definitions of maternal complications**

Pregnancy complications (start before labour onset)

− Maternal anaemia: severe anaemia

− Maternal diabetes: diabetes mellitus

− Preeclampsia/eclampsia: (high blood pressure during the last three months of pregnancy and blurred vision) or convulsions during pregnancy

− Antepartum haemorrhage: Any vaginal bleeding in the last three months of pregnancy

Labour/delivery complications (start after labour onset)

− Maternal anaemia: same as for pregnancy

− Preeclampsia/eclampsia: (High blood pressure during labour/delivery and blurred vision) or (convulsions during labour/delivery and no fever during labour)

− Prolonged rupture of membranes: Water broke 24/more hours before baby was born

− Fever during labour: fever during labour

− Maternal infection: Fever during labour and (foul smelling amniotic fluid or foul-smelling vaginal discharge during delivery)

− Prolonged labour: Labour lasted 12/more hours

− Malpresentation: newborn delivered not head first

− Cord complication: Cord delivered first or around the newborn’s neck more than once

### Statistical analyses

We used Fisher’s exact test to assess the association of maternal complications with: providers/facilities where mothers sought care on route to their DF, the timing of neonates’ death before or after discharge from their birth facility, and the likelihood of neonates’ referral from the birth facility. We used the χ^2^ test of proportions to examine the association of maternal complications with the place where neonates’ fatal illnesses began and, for neonates delivered in a facility, with the timing of death in relation to discharge from the facility. We used the *t* test of equivalence of means to evaluate the number of providers/facilities where women sought care for their L/D complications on route to their DF and the Wilcoxon two-sample test with normal approximation of Z to assess their delays 1 and 2, as well as to explore the association of neonates’ age at illness onset and death with place of illness onset. We examined skewness and kurtosis of test samples to assess normality, with acceptable limits from -1 to 1, to determine whether to use the Wilcoxon or *t* test to evaluate significance (at *P* < 0.05). We developed logistic regression models to examine variables associated with delivery place (DH, LLDF, community) and delivery place by onset of neonates’ fatal illness. Possible predictor variables included any maternal complication (yes, no), mothers’ education level (primary, secondary, higher), insurance coverage (yes, no), ANC attendance (four or more visits (ANC4+), less than four), and time to reach the nearest health facility in an emergency (less than 30 minutes, 30 minutes or more). Cases with missing data for any variable were excluded from analyses.

### Modified Pathway to Survival

To account for the finding that many neonates died in their DF before discharge, we modified the P-to-S graphic to incorporate women’s care-seeking for their complications, delivery place, and place of neonates’ illness onset.

## RESULTS

### Study population

The NODE-IN/NODE-FOR process yielded 902 potential neonatal deaths or stillbirths, of which 272 (30.2%) were confirmed as study-eligible neonatal deaths. During home visits, 31.2% cases were ineligible due to being stillbirths, 21.9% due to having occurred outside of the study period, 12.5% due to having a gestational age <28 weeks, 2.2% due to being >27 days old at death, and 2.0% due to either being duplicate cases or having unconfirmable eligibility. Just over 95% (259/272) of eligible cases had a complete VASA interview, so we included them in the analysis. Among these, the mean recall period since death was 5.4 (standard deviation (SD) = 2.0) months.

To assess the completeness of our study sample, we compared the 272 confirmed neonatal deaths to official health system records. The 272 deaths comprised 1.6 and 2.2 times more neonatal deaths than reported by Serang and Jember district records, respectively; and, in total, the 272 deaths comprised 92.3% of all neonatal deaths captured by NODE-IN/NODE-FOR and the health system records.

Almost 80% of the 259 neonatal deaths with a complete VASA interview died within one week of birth (**Table 1**). Altogether, 215 (83.0%) and 191 (73.7%) of 259 neonates had been born and had died in a health facility, respectively; 114 (44.0%) had been born and had died in the same facility without leaving, including 91 (75.2%) of 121 hospital births and 23 (24.5%) of 94 lower-level facility (LLF) births. The odds of either a neonate born in a hospital vs a neonate born elsewhere or a neonate born in a LLF vs a neonate born elsewhere dying in their birth facility without leaving were 15.2 (95% confidence interval (CI) = 8.3-27.9) and 0.3 (95% CI = 0.2-0.5), respectively.

**Table 1:** Characteristics of mothers and newborns

Characteristic	Neonates or mothers, n (%)
**Neonate’s age (days)**	
0	88 (34.0)
1-6	116 (44.8)
7-13	30 (11.6)
14-27	25 (9.7)
**Neonate’s sex**	
Male	157 (60.6)
Female	102 (39.4)
**Birth place**	
Hospital	121 (46.7)
LLDF	94 (36.3)
Home	37 (14.3
On route to a facility	7 (2.7)
**Neonate’s place of death**	
Hospital	163 (62.9)
LLDF	28 (10.8)
Home	63 (24.3)
On route to a facility	5 (1.9)
**Mother’s age (years)**	
<20	27 (10.4)
20-29	109 (42.1)
≥30	123 (47.5)
Mean (SD) age	29.2 (7.6)
Mother’s schooling*	
None	3 (1.2)
Primary	84 (32.4)
Secondary	156 (60.2)
Higher	15 (5.8)
Missing	1 (0.4)

LLDF – lower-level delivery facility, SD – standard deviation

*Highest level attended.

We found no difference in the ages of the 215 mothers who delivered in a health facility and 44 who delivered in the community (29.1 vs 29.8; *P* = 0.57), nor in the ages of the 189 mothers whose neonate’s fatal illness started in a health facility and 70 whose neonate’s fatal illness started in the community (29.0 vs 29.9, *P* = 0.41). We also found no difference in the schooling level of mothers who delivered in a health facility and the community (chi-square (χ^2^) = 4.27; *P* = 0.12), nor of mothers whose neonate’s fatal illness began in a health facility and the community (χ^2^ = 3.78; *P* = 0.15).

### Delivery place, illness onset and death

**Table 2** shows the age at illness onset and at death of the 259 neonates, according to their location at onset and when their fatal illness began. Illness onset for groups one through three was in the community, while for groups four and five illness began in the LLDF and DH, respectively. Illness started at a younger age and death occurred earlier for neonates whose illness started at their DF vs in the community. The earlier illness onset of neonates in their LLDF, compared to neonates at their DH, was due to five DH outliers whose illness started at 13 days of age.

**Table 2:** Age at onset of fatal illness and death of neonates, by place of illness onset

n	Place where: when neonate’s fatal illness began	Age (days) when fatal illness started								Age (days) at death							
		**n**	**Mean (95% CI)**	**Median (IQR)**	*P*-value*	**n**	**Mean (95% CI)**	**Median (IQR)**	*P*-value*	**n**	**Mean (95% CI)**	**Median(IQR)**	*P*-value*	**n**	**Mean (95% CI)**	**Median (IQR)**	*P*-value*
35	Community: during/after delivery without an SBA	35	1.9 (0.4-3.5)	0 (0-1)		70	3.6 (2.1-5.2)	0 (0-4)	<0.001	35	3.5 (1.6-5.4)	0 (0-7)		70	5.3 (3.6-7.1)	2 (0-7)	0.06
6	Community: during delivery with an SBA	6	0 (0-0)	0 (0-0)						6	1.7 (0.6-2.8)	2 (1-2)					
29	Community: after healthy delivery in the community with an SBA (3) or at a facility (26)	29	6.5 (3.3-9.7)	3 (0-9)						29	8.3 (4.9-11.8)	3 (2-12)					
74	LLDF: during/after delivery, before discharge	73	0 (0-0)	0 (0-0)	0.01	188	0.3 (0.06-0.5)	0 (0-0)		74	2.7 (1.7-3.7)	1 (0-2)	0.25	189	3.5 (2.7-4.3)	1 (0-4)	
115	DH: during/after delivery, before discharge	115	0.5 (0.1-0.8)	0 (0-0)						115	4.0 (2.8-5.1)	1 (0-5)					

CI – confidence interval, IQR – interquartile range, SBA – skilled birth attendant, LLDF – lower-level delivery facility, DH – hospital-delivery

*From Wilcoxon 2-sample test with normal approximation of Z.

### Maternal complications, delivery place and illness onset

**Table 3** shows the mothers’ maternal complications by the same categories of neonate’s place of illness onset as displayed in **Table 2**. The most common complications were prolonged labour, malpresentation, and PROM. Mothers whose neonate’s fatal illness started in their DF, whether a hospital, LLDF or any facility, had more complications than mothers whose neonate’s illness began in the community; as did mothers whose neonate’s illness started in their DH, compared to those whose neonate’s illness began in their LLDF.

**Table 3:** Maternal complications, by place where fatal neonatal illness began

N of deaths	Place where: when neonate’s fatal illness began	Complication								OR (95% CI)			
		**Precl/ecl, n (%)**	**PROM, n (%)**	**APH, n (%)**	**Fever during labour, n (%)**	**Prolonged labour, n (%)**	**Mal-presentation, n (%)**	**Cord complication, n (%)**	**Any complication, n (%)**	**Illness began at DH vs community**	**Illness began at DH or LLDF vs community**	**Illness began at DH vs LLDF**	**Illness began at LLDF vs community**
35	Community: during/after delivery without an SBA	0 (0)	1 (3)	0 (0)	1 (3)	4 (11)	5 (14)	1 (3)	9 (26)				
6	Community: during delivery with an SBA	0 (0)	0 (0)	0 (0)	0 (0)	1 (17)	0 (0)	1 (17)	2 (33)				
29	Community: after healthy delivery in the community with an SBA (3) or at a facility (26)	0 (0)	0 (0)	3 (10)	2 (7)	3 (10)	2 (7)	2 (7)	9 (31)				
74	LLDF: during/after delivery, before discharge	1 (1)	6 (8)	1 (1)	2 (3)	21 (28)	7 (9)	2 (3)	33 (45)		4.0 (2.2-7.2)*		2.0 (1.0-4.0)†
115	DH: during/after delivery, before discharge	5 (4)	12 (10)	11 (10)	11 (10)	52 (45)	18 (16)	3 (3)	83 (72)	6.5 (3.4-12.5)*		3.2 (1.7-6.0)*	

OR – odds ratio, CI – confidence interval, Precl/ecl – preeclampsia/eclampsia during last three months of pregnancy or labour/delivery, PROM – prolonged rupture of membranes, APH – antepartum haemorrhage, SBA – skilled birth attendant, DH – delivery hospital, LLDF – lower-level delivery facility

*P < 0.001.

†*P* = 0.046.

**Table 4** shows the same complications for mothers of neonates whose illness started in their DF, but additionally categorized by whether the neonate died before leaving the facility or after being discharged alive. Mothers whose neonate died before leaving their DH were 2.8 times more likely to have a complication than mothers whose baby died after discharge. There was no such excess for women whose newborn’s fatal illness began in their LLDF and died there without leaving. There were also differences within and between the two groups of women in complication types, with PROM, preeclampsia/eclampsia, APH and malpresentation more common among women whose neonates died in their DH before discharge than among those whose neonates left alive; and, nearly so, the opposite pattern for LLDF deaths.

**Table 4:** Maternal complications, by delivery facility level where fatal neonatal illness began and timing of death

N of deaths	Facility level where neonate was delivered and fatal illness began: timing of death	Complication							Any complication, n (%)	OR (95% CI)	*P*-value*	Any of four complications, n (%)†	OR, (95% CI)	*P*-value
		**Pre/ecl, n (%)**	**PROM, n (%)**	**APH, n (%)**	**Fever during labour, n (%)**	**Prolonged labour, n (%)**	**Malpresentation, n (%)**	**Cord complication, n (%)**						
51	LLDF: left alive	1 (2)	5 (10)	1 (2)	0 (0)	14 (27)	6 (12)	2 (4)	23 (45)	0.9 (0.3-2.5)	0.90	13 (57)	0.2 (0.03-1.1)	0.07‡
23	LLDF: died before discharge	0 (0)	1 (4)	0 (0)	2 (9)	7 (30)	1 (4)	0 (0)	10 (43)			2 (20)		
24	DH: left alive	0 (0)	0 (0)	0 (0)	2 (8)	10 (42)	2 (8)	1 (4)	13 (54)	2.8 (1.1-7.2)	0.03	2 (15)	6.9 (1.4-33.6)	0.01*
91	DH: died before discharge	5 (5)	12 (13)	11 (12)	9 (10)	42 (46)	16 (18)	2 (2)	70 (77)			39 (56)		

Pre/ecl – preeclampsia/eclampsia during last three months of pregnancy or labour/delivery, PROM – prolonged rupture of membranes, APH – antepartum haemorrhage, OR – odds ratio, CI – confidence interval, LLDF – lower-level delivery facility, DH – delivery hospital

*By χ^2^ test of proportions.

†Preeclampsia/eclampsia, prolonged rupture of membranes, antepartum haemorrhage, or malpresentation among women with any complication.

^‡^By Fisher’s exact test.

Most neonates discharged alive were referred, including 41/51 (80.4%) LLDF deliveries and 16/24 (66.7%) DH deliveries; LLDF neonates were referred more often (70.3% vs 26.1%; *P* < 0.001) and earlier (mean age 4.0 vs 43.9 hours; *P* = 0.01). Maternal complications were not associated with neonates’ referral from LLDFs (24/33 (72.7%) with vs 28/41 (68.3%) without a complication; *P* = 0.68) or DHs (21/83 (25.3%) with vs 9/32 (28.1%) without a complication, *P* = 0.76). Almost all neonates, equally among those whose mother did and did not have a complication, were referred due to a lack of equipment, medicines, procedures and/or trained providers needed to deliver quality newborn care vs insurance or other matters (LLDFs: 21/24 (87.5%) with vs 24/27 (88.9%) without a complication; *P* = 1.00; DHs: 20/21 (95.2%) with vs 8/9 (88.9%) without a complication; *P* = 0.52). All known (26/30) referrals from a DH, and all but four of 50 known referrals from a LLDF, were to a hospital.

### Factors associated with delivery place

When adjusted for other potential explanatory factors, DH delivery was strongly associated with having one or more maternal complications and any insurance coverage (**Table 5**). Delivery in any facility was associated with the same factors, but the significant factors for LLDF delivery were ANC4+, insurance coverage, and travel time. Analyses of neonates whose illness began in their DF yielded similar results, but with DH delivery even more strongly associated with maternal complications (adjusted odds ratio (aOR) = 4.0; 95% CI = 2.3-6.9); and LLDF delivery no longer significantly related to ANC4+ (aOR = 2.7; 95% CI = 0.8-8.9).

**Table 5:** Unadjusted and logistic regression-adjusted analyses of factors possibly associated with delivering in a hospital and a lower-level facility*

Explanatory factors	Hospital delivery vs all others						Lower-level facility delivery vs community delivery					
	**DH, n (%)**	**All others, n (%)**	**OR (95% CI)**	***P*-value**	**aOR (95% CI)**	***P*-value**	**LLDF, n (%)**	**Community, n (%)**	**OR (95% CI)**	***P*-value**	**aOR (95% CI)**	***P*-value**
**Maternal complication†**				<0.001		<0.001				0.11		
No complication (reference)	36 (29.8)	87 (63.0)	–	–	–	–	55 (58.5)	32 (72.7)	–	–	–	–
1 or more complications	85 (70.2)	51 (37.0)	4.0 (2.4-6.8)	–	3.6 (2.1, 6.2)	–	39 (41.5)	12 (27.3)	1.9 (0.9-4.1)	–	–	–
**Antenatal care visits**				0.78						0.03		0.03
Less than 4 visits (reference)	11 (9.2)	14 (10.2)	–	–	–	–	6 (6.4)	8 (18.6)	–	–	–	–
4 or more visits	109 (90.8)	123 (89.8)	1.1 (0.5-2.6)	–	–	–	88 (93.6)	35 (81.4)	3.4 (1.1-10.4)	–	3.9 (1.2-13.3)	–
**Insurance coverage‡**				<0.001		<0.001				0.02		0.03
No insurance (reference)	29 (24.0)	68 (49.3)	–	–	–	–	40 (42.6)	28 (63.6)	–	–	–	–
Any insurance coverage	92 (76.0)	70 (50.7)	3.1 (1.8-5.3)	–	2.7 (1.5, 4.7)	–	54 (57.4)	16 (36.4)	2.4 (1.1-4.9)	–	2.4 (1.1-5.2)	–
**Mother’s schooling**				0.49						0.14		
None/any primary school (reference)	35 (28.9)	52 (38.0)	–	–	–	–	31 (33.0)	21 (48.8)	–	–	–	–
Any secondary school	79 (65.3)	77 (56.2)	1.5 (0.9-2.4)	–	–	–	56 (59.6)	21 (48.8)	1.5 (0.7-3.2)	–	–	–
Any higher level	7 (5.8)	8 (5.8)	1.0 (0.3-2.8)	–	–	–	7 (7.4)	1 (2.3)	3.4 (0.4-28.4)	–	–	–
**Travel time to nearest health facility in an emergency**				0.60						0.002		0.004
One-half hour or more (reference)	14 (11.6)	19 (13.8)	–	–	–	–	7 (7.4)	12 (27.3)	–	–	–	–
Less than one-half hour	107 (88.4)	119 (86.2)	1.2 (0.6-2.6)	–	–	–	87 (92.6)	32 (72.7)	4.7 (1.7-12.9)	–	4.8 (1.7-14.0)	–

DH – delivery hospital, OR – odds ratio, CI – confidence interval, aOR – adjusted odds ratio, LLDF – lower-level delivery facility

*All factors with univariate *P* < 0.20 allowed to enter the logistic regression models.

†Maternal complications include preeclampsia/eclampsia, prolonged rupture of membranes, antepartum haemorrhage, fever during labour, prolonged labour, foetal malpresentation and/or cord complication.

‡Insurance coverage includes National Health Insurance (NHI), government beneficiaries not covered by NHI, employer-provided and other private insurance.

### Care-seeking delays for maternal complications

**Figure 1:**
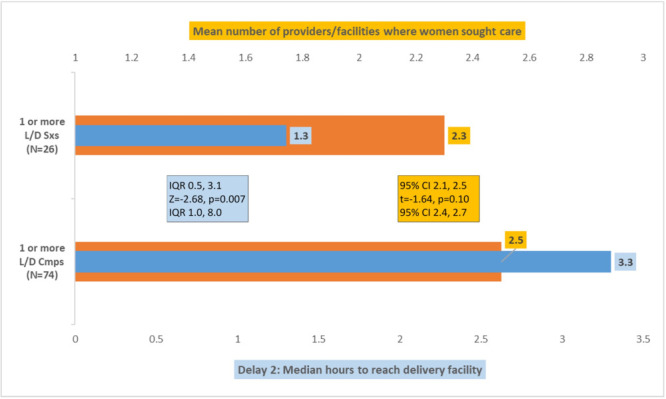
100 women who sought care from at least one other provider/facility on route to their delivery facility. L/D Sxs – labour/delivery symptoms not meeting the criteria for a complication, L/D Cmps – labour/delivery complications, IQR – interquartile range, CI – confidence interval.

Among 172 women with one or more L/D symptoms who delivered in a facility, 100 sought care from at least one other provider or facility on route to their DF. There was no difference in delay 1 (0.08 vs 0.03 hours; *P* = 0.62) for the 74/100 women with a L/D complication and 26 with symptom(s) that did not meet the box 1 criteria for a complication. However, while the 100 women went to the same number of providers/facilities, those with a complication took significantly longer to reach their DF than those without a complication (**Figure 1**). The symptoms of 89 of these 100 women started in the community without a skilled birth attendant (SBA). Providers/facilities they visited most often before reaching their DF included hospitals (28.4% of women with a complication vs 0.0% without; *P* = 0.003) (all of whom delivered at a hospital), puskesmas (25.4% vs 18.2%; *P* = 0.57) and private midwives (20.9% vs 18.2%; *P* = 1.00). In contrast to these 100 women, there was no difference in arrival time between the 22 women with and 50 without a complication (median = 0.3 vs 0.2 hours; *P* = 0.59) who went directly to their DF.

One hundred ten of the 172 women delivered in a hospital and drove the findings for the total 172 women: of the 110 women, 79 sought care from at least one other provider/facility on route to their DH, of whom the 62 with a L/D complication took the same 3.3 hours to reach their DH as all 74 women with a complication took to reach their DF. These 62 women and the 17 without a complication went to the same number of providers/facilities on route to their DH (mean = 2.6 vs 2.4; *P* = 0.41), and there was some indication that those with a complication took almost one hour longer to reach their DH, but this was not statistically significant (median = 3.3 vs 2.5 hours; *P* = 0.12). There was no difference in delay 1 (median = 0.08 vs 0.17 hours; *P* = 0.28) for these 62 and 17 women. Of the 31/110 women who went directly to their DH, 23 with and eight without a complication took the same time (0.0 vs 0.02 hours; *P* = 0.74) to reach it.

Among the 62 women who delivered in a LLDF, 12 with and nine without a complication who sought care from at least one provider/facility on route to their LLDF went to 2.3 and 2.0 facilities (*P* = 0.19) and reached their LLDF in 0.9 and 0.5 hours (*P* = 0.29), respectively, while the 27 women with and 14 without a complication who went directly to their LLDF arrived in 0.3 and 0.4 hours (*P* = 0.72), respectively.

In total, the 110 women who delivered at DH went to more facilities than the 62 women who delivered at LLDF (mean = 2.1 vs 1.4; *P* < 0.001), and they took longer to reach their DF (median = 2.0 vs 0.5 hours; *P* < 0.001).

Having National Health Insurance (NHI) coverage did not affect whether women with and without a L/D complication sought delivery care at multiple providers/facilities (NHI: 35/54 (64.8%) with vs 12/23 (56.5%) without a complication, *P* = 0.49; no NHI: 39/70 (55.7%) with vs 13/25 (52.0%) without a complication, *P* = 0.75).

### Modified Pathway to Survival

**Figure 2:**
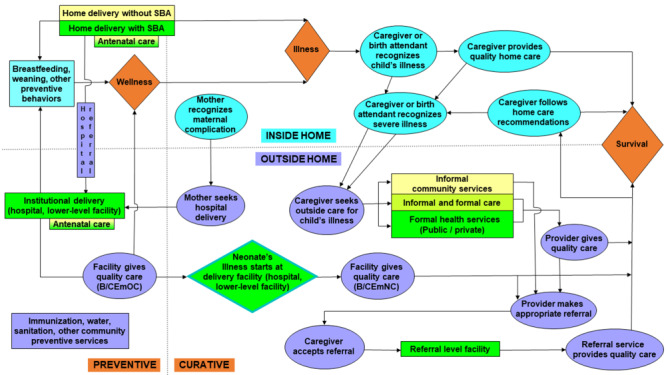
Modified Pathway to Survival. Colour and shape key: orange diamond = health condition/outcome; blue rectangle/oval = inside-the-home preventive care, illness recognition and care provision; purple rectangle/oval = outside-the-home preventive care, care-seeking and health care provision; yellow rectangle = informal care; light green rectangle = mixed informal and formal health care providers/facilities; bright green rectangle/diamond = formal health care provider/facility. B/CEmOC – Basic and Comprehensive Emergency Obstetric Care (to prevent neonatal illness), B/CEmNC – Basic and Comprehensive Emergency Neonatal Care (to treat neonatal illness).

**Figure 2** presents our modified P-to-S, which adds maternal antenatal and delivery care to the left side of the original Pathway, and recognition and care-seeking for maternal complications and emergency neonatal care to the right side.

Our companion study [30] focused on care-seeking for sick neonates, including those whose illnesses started in the community. Following the modified Pathway’s maternal care steps provides a summary of the findings discussed in detail above. Nearly all (90%) of the 259 women achieved ANC4+, including 109 (91%) who delivered at DH, 88 (94%) who delivered at LLDF, and 35 (81%) women who delivered in the community. One hundred thirty-six (53%) of the women had a maternal complication that they were able to report at interview; 85 (63%) delivered at DH, 39 (29%) at LLDF, and 3 (1%) and 9 (3%) delivered with and without an SBA in the community. Of 85 babies delivered at a hospital whose mother had a complication, the fatal illnesses of 83 (98%) began in a hospital and two (2%) went home healthy; 70 (84%) of the 83 died before discharge, and 13 (16%) left alive, 10 (77%) of whom were referred to another hospital (n = 9/10) or unknown facility (n = 1/10). Of 39 babies delivered at LLDF whose mother had a complication, the fatal illnesses of 33 (85%) began in the LLDF and six (15%) went home healthy; 10 (30%) of the 33 died before discharge and 23 (70%) left alive, 20 (87%) of whom were referred to a hospital (n = 17/20) or other LLDF (n = 3/20). Two (67%) of the three babies delivered by an SBA in the community whose mother had a complication were sick at birth; one was referred to a hospital and the other one to a LLDF. We did not collect referral data for babies of women who delivered without an SBA.

## DISCUSSION

Indonesia’s roadmap to attaining and exceeding its Countdown to 2030 maternal and neonatal mortality reduction targets [34] lies in ensuring that all births take place in a BEmONC- or CEmONC-capable facility and that, in line with Indonesian government policy, all women with complications deliver in a CEmONC facility [35]. Indeed, we found a strong connection between maternal complications and delivery place, especially hospital delivery. More than 70% of neonates’ fatal illnesses began in their birth facility, and more than 60% of these neonates’ mothers had one or more complications, more than twice the rate of mothers who delivered at home and nearly twice as great in DH- vs LLDF-deliveries. However, this was most often associated with EN illness onset, on average at several hours of age for facility deliveries that were ill at birth vs nearly four days for neonates whose illnesses began in the community; and facility onset led to death in three and a half days compared to more than five days for community-onset illnesses. While most women with a complication delivered at a hospital, delays related to visiting multiple providers and facilities before reaching the DH may have contributed to the deaths.

Nearly 80% of the deaths identified by the EMNC study were of ENs, the deaths most likely to have a maternal underlying cause [3,7]. Also, nearly 80% of neonates whose illness started in their DH and 30% of those whose illness started in their LLDF died before leaving; mothers of neonates who died in their DH were more likely to have a complication than those whose neonates left their DH alive, and their complications were also more likely to differ, with preeclampsia, PROM, APH, and malpresentation being most problematic. These findings again suggest a strong connection between maternal complications, as well as the types of complications, and neonatal illness severity. Other studies in low- and middle-income countries (LMIC) have also found malpresentation [7,36], hypertensive disorders [7,37] and APH [37,38] to be strongly associated with EN death.

Among LLDF-delivered neonates, these complications were nearly significantly more common among those discharged alive, although we did not find an association between maternal complications and neonatal referral either from LLDF or DH. Nevertheless, the higher referral rate and earlier referral of neonates from LLDF suggest a decreased capability of LLDFs to care for these sick newborns. Hospitals and LLDFs faced the same shortcomings in quality of care indicators that mothers, both those with and without complications, reported necessitated referral of their newborns, although, given their higher referral rate, apparently at greater levels in LLDFs. These findings suggest that many women delivered at facilities incapable of providing emergency obstetric and neonatal care.

Multivariable analysis of delivery place confirmed the strong association of maternal complications with hospital delivery, and a weaker association with LLDF delivery of sick neonates. Women’s knowledge of intrapartum danger signs [39] and the presence of complications during labour [40] have previously been shown to increase institutional delivery. Insurance coverage and travel time were stronger predictors of facility delivery for women who delivered a sick neonate in an LLDF; while insurance coverage also promoted hospital delivery for all women and women whose neonate’s illness began in the DH. Other studies have shown a positive effect of NHI coverage on institutional delivery in Indonesia [41,42]. To our best knowledge, this is the first study to examine the effect of any insurance coverage, including private insurance and government assistance to beneficiaries not covered by NHI, on facility delivery. ANC4+ has also been previously shown to increase institutional delivery in Indonesia [16,41] and elsewhere [43,44]. We found ANC4+ to be associated with LLDF- but not DH-delivery, and not in women whose neonate’s fatal illness began in the LLDF. These findings suggest that rapid access, including distance and cost considerations, took precedence over the benefit incurred from ANC visitation in women with maternal complications.

Rapid access to delivery care is especially critical in the case of a woman in labour with a complication. Women in the EMNC study with and without L/D complications took the same amount of time to decide to seek care and went to the same number of providers/facilities before reaching their DF, yet those with complications more frequently went to a hospital on route and took on average up to two hours longer to reach their DF. Such delays were greater in women who delivered at a hospital, suggesting their need for more care before being discharged or referred and potentially more severe outcomes as the result of any delay in accessing definitive care. Although having any insurance coverage increased facility delivery, NHI coverage did not decrease women’s seeking care for their L/D complications at multiple providers/facilities on route to their DF, despite NHI policy that women with emergency complications should go directly to a hospital without a need for referral [45]. An increased delay in reaching appropriate care for maternal complications and neonatal illnesses due to multiple referrals has also been found in other settings [46].

### Modified Pathway to Survival

This study and a companion study of care-seeking for 259 neonatal deaths along the P-to-S identified the need for a modified Pathway that recognizes the importance of rapid access to quality institutional delivery care in settings where many births occur in facilities and/or there is good care-seeking for L/D complications. This is the case in many LMICs, as other studies have found an increase in institutional births accompanied by shifting of high-risk births with maternal complications to facilities [47,48]. The original P-to-S (Figure S1 in the **Online Supplementary Document**) was designed more than a quarter century ago as a program development and evaluation tool in support of the WHO/UNICEF IMCI approach of promoting wellness, illness recognition and care-seeking for one-week to 59-month-old children whose illnesses started at home [25]. The IMCI chart booklet was updated in 2014 to include care of ENs [49], but the Pathway has not been updated accordingly. With the epidemiological transition to neonatal death as the main driver of U5 mortality, a modified P-to-S is needed that examines inadequate access to quality BEmONC and CEmONC for all parturient women and especially those with maternal complications. Other authors have depicted pathways aimed at newborn survival [50,51], but none that incorporate all the elements of the original P-to-S. The modified Pathway that we present preserves and builds on the original, and should prove a useful supporting tool in the development and monitoring of interventions against neonatal and child mortality that recognize the importance of antenatal and delivery care, care-seeking for maternal complications, and provision of quality BEmONC and CEmONC for women and newborns.

The modified Pathway includes steps at which delays in deciding to seek health care and reaching appropriate care both for women with complications and sick children can be assessed. The Three Delays model for examining the contribution to maternal mortality of these two delay types plus the delay in the provision of quality care once reaching an appropriate facility was formulated nearly three decades ago [52], and has since been used in multiple studies of maternal death and adapted to assess delays contributing to NM [53]. However, the model has seldom (and, to our knowledge, never before in Indonesia) been applied to evaluate the impact of delays in care-seeking for maternal complications on neonatal death [28] or stillbirth [54]. Assessment of, and determining the reasons for, these delays at key steps in the Pathway will make it an even more powerful supporting tool in the fight against NM.

### Limitations

All the study data are from interview reports, mainly of the mothers of the deceased neonates, so are subject to possible recall and reporting biases. However, the recall period was shorter than for many VASA studies, which should have minimized recall bias. We defined algorithms of maternal complications based on reports of individual obstetric illness signs and symptoms in the 2016 WHO VA questionnaire, which for some conditions are somewhat limited. For example, hypertension, blurred vision, and convulsions are the only symptoms available with which to construct an algorithm for preeclampsia/eclampsia. Therefore, we may have underestimated the proportion of women with some complications, but this should not have affected the relative levels estimated for women who delivered at a facility and in the community. Small sample size for some sub-group analyses limits our definitiveness in drawing certain conclusions, for example, that neonates whose mothers had particular complications were more likely than those whose mothers had other complications to be discharged alive from their LLDF.

The VASA study was not designed to assess the quality of care provided by delivery facilities, and we could not determine which facilities were capable of providing BEmONC and CEmONC. Increased access to institutional delivery by itself, in the absence of improvements in the quality of care, will not decrease NM and in some instances may even have a detrimental effect [48]. Our findings, that a major portion of neonatal deaths were related to maternal complications and subsequent newborn illnesses cared for in delivery facilities, call for a follow-up study to directly assess the quality of care provided for L/D complications and neonatal illnesses.

## CONCLUSIONS

Most neonatal deaths in two districts of Java, Indonesia were of infants whose fatal illnesses started on the day of birth at their DF. Most of these newborns’ mothers had one or more maternal complications, and many mothers with a L/D complication visited multiple providers/facilities before reaching their DF, leading to delays that might have contributed to their newborn’s death. Given the global trend toward increasing institutional delivery, especially in LMICs, and the increased share of neonatal deaths due to ENM, ANC providers should instruct women about complications for which they should go directly to a CEmONC-capable hospital for delivery; and governmental and institutional policy should support women in following these instructions. A modified Pathway to Survival highlights the increased importance of rapid access to quality institutional delivery care in settings where many births occur in facilities and/or there is good health care-seeking for L/D complications. Health systems in LMICs should consider incorporating use of the modified P-to-S into their ongoing maternal, neonatal and child health program development and monitoring activities.

## Additional material


Online Supplementary Document
