# Supplementary material for: Modified Pathway to Survival highlights importance of rapid access to quality institutional delivery care to decrease neonatal mortality in Serang and Jember districts, Java, Indonesia
Source: J Glob Health. 2023 Apr 14;13:04020. doi: 10.7189/jogh.13.04020 (PMC10101726; doi:10.7189/jogh.13.04020)

Figure S1. The original Pathway to Survival. Color and shape key: orange diamond=health condition/outcome; blue rectangle/oval=inside-the-home preventive care, illness recognition and care provision; purple rectangle/oval=outside-the-home preventive care, care seeking and health care provision; yellow rectangle=informal care; light green rectangle=mixed informal and formal health care providers/facilities; bright green rectangle=formal health care provider/facility.

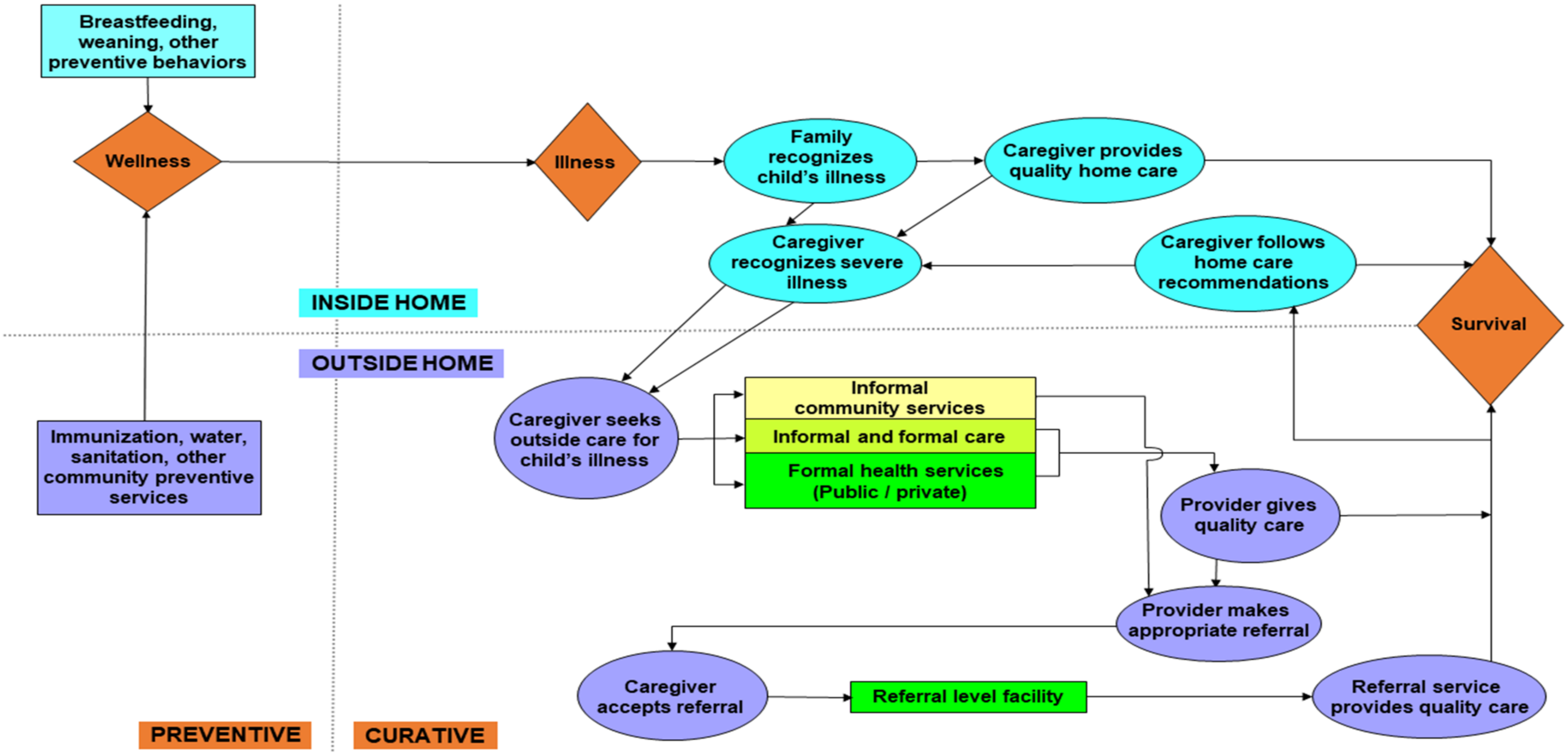

Supplement: Online Supplementary Document [file jogh-13-04020-s001.pdf]
